# Changes and drivers of zooplankton diversity patterns in the middle reach of Yangtze River floodplain lakes, China

**DOI:** 10.1002/ece3.8353

**Published:** 2021-12-15

**Authors:** Quanfeng Lu, Xiongjun Liu, Xuemei Qiu, Tao Liang, Jinping Chen, Shuai Zhao, Shan Ouyang, Binsong Jin, Xiaoping Wu

**Affiliations:** ^1^ School of Life Sciences Nanchang University Nanchang China; ^2^ Guangdong Provincial Key Laboratory of Conservation and Precision Utilization of Characteristic Agricultural Resources in Mountainous Areas School of Life Science Jiaying University Meizhou China; ^3^ Jiangxi Province Key Laboratory of Watershed Ecosystem Change and Biodiversity School of Life Sciences Nanchang University Nanchang China

**Keywords:** community structure, floodplain lakes, species diversity, Yangtze River, zooplankton

## Abstract

Anthropogenic habitat alteration interferes the natural aquatic habitats and the system's hydrodynamics in the Yangtze River floodplain lakes, resulting in a serious decline in freshwater biodiversity. Zooplankton communities possess major position in freshwater ecosystems, which play essential parts in maintaining biological balance of freshwater habitats. Knowledge of processes and mechanisms for affecting variations in abundance, biomass, and diversity of zooplankton is important for maintaining biological balance of freshwater ecosystems. Here, we analyzed that the temporal and spatial changes in the structure of zooplankton community and their temporal and spatial variations respond to changes in environmental factors in the middle reach of Yangtze River floodplain lakes. The results showed that zooplankton samples were classified into 128 species, and Rotifera was the most common taxa. Significant seasonal differences were found among the abundance and diversity of zooplankton. Similarly, we also found significant seasonal differences among the biomass of zooplankton functional groups. The spatial turnover component was the main contributor to the β diversity pattern, which indicated that study areas should establish habitat restoration areas to restore regional biodiversity. The NMDS plot showed that the structure of zooplankton community exhibited significant seasonal changes, where the community structure was correlated with pH, water temperature, water depth, salinity, total nitrogen, chlorophyll‐*a*, and total phosphorus based on RDA. This study highlights that it is very important to ensure the floodplain ecosystem's original state of functionality for maintaining the regional diversity of the ecosystem as a whole.

## Graphical abstract

Anthropogenic habitat alteration interferes the natural aquatic habitats and the system's hydrodynamics in the Yangtze River floodplain lakes, resulting in a serious decline in freshwater biodiversity. Zooplankton communities possess major position in freshwater ecosystems, which play essential parts in maintaining biological balance of freshwater habitats. Knowledge of processes and mechanisms for affecting variations in density, biomass, and diversity of zooplankton is important for maintaining biological balance of freshwater ecosystems. Here, we analyzed that the temporal and spatial changes in the structure of zooplankton community and their temporal and spatial variations respond to changes in environmental factors in the Yangtze River floodplain lakes. The results showed that zooplankton samples were classified into 128 species, and Rotifera was the most common taxa. Significant seasonal and spatial differences were found among the density, biomass, and diversity of zooplankton. Similarly, we also found significant seasonal and spatial differences among the biomass of zooplankton functional groups. The spatial turnover component was the main contributor to the beta diversity pattern, which indicated that the study areas should establish habitat restoration areas to restore regional biodiversity. The NMDS plot showed that the structure of zooplankton community exhibited significant seasonal changes, where the community structure was significantly correlated with pH, water temperature, water depth, salinity, total nitrogen, chlorophyll‐*a*, and total phosphorus based on RDA. This study highlights that it is very important to ensure the floodplain ecosystem's original state of functionality for maintaining the regional diversity of the ecosystem as a whole.

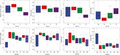

## 1 INTRODUCTION

Freshwater ecosystems in the past few decades are heavily affected by anthropogenic disturbances, such as habitat degradation, dam construction, water pollution, and species invasion, which have put these natural environments at risk and affected biodiversity (Agostinho et al., 2009; Azan et al., 2015; Dudgeon et al., 2006). For example, in the floodplains, anthropogenic habitat alteration interferes the natural aquatic habitats and the system's hydrodynamics (Agostinho et al., 2004), resulting in a serious decline in freshwater biodiversity (Dudgeon, 2019; Dudgeon et al., 2006). To protect freshwater habitat, it is important to know the impact of different stressors on diversity, which provide key information on the temporal variation of diversity and the mechanisms by which ecosystems are altered, especially under intense anthropogenic disturbance (Altshuler et al., 2011; Dudgeon, 2019).

Zooplankton plays a major role in freshwater ecosystems, and in the food webs, linking primary producers and consumers (Compte et al., 2016; Keister et al., 2012; Varadharajan & Soundarapandian, 2013; Waidi et al., 2016). The functions performed by zooplankton in the freshwater ecosystem are closely related to ecosystem services (Dornelas et al., 2013), which is indispensable for the maintenance of human life. Zooplankton species can test hypotheses about the diversity variation through different spatial scales (Declerck et al., 2011; Melo & Medeiros, 2013). Zooplankton are highly diverse and abundant in floodplain lakes, which have different functional traits, and their distribution is influenced by environmental filters (Bozelli et al., 2015; Chaparro et al., 2011; Simões et al., 2013). Due to zooplankton with different niche requirements (Allan, 1976; Bonecker et al., 2009; Bozelli et al., 2000), this community is used with investigating the impact of local factors on the community structure of floodplain lakes. Some studies showed the use of zooplankton communities as a useful tool to aid in the establishment of priority areas for conservation (Dodson et al., 2009; Louette et al., 2008; Palmer et al., 2013). Therefore, zooplankton communities possess major position in freshwater ecosystems, which play essential parts in maintaining biological balance of freshwater habitats (Loick‐Wilde et al., 2016).

Species interactions are complex in floodplain ecosystems because periodic changes in the environmental characteristics of lakes and flood pulses (Bini et al., 2001; Górski et al., 2013; Lansac‐Tôha et al., 2009; Simões et al., 2013). The dry periods can increase species differentiation of zooplankton among areas in response to changes in environmental conditions (Bozelli et al., 2015; Velho et al., 2004), whereas the floods can enhance biological homogenization among areas (Alves et al., 2005; Simões et al., 2013). In floodplain lakes, some studies showed that variation in zooplankton community was strongly related to flood pulse and environmental heterogeneity (Bozelli et al., 2015; Górski et al., 2013). The Yangtze River floodplain is one of the world's largest floodplains, containing about 3% of all freshwater fish species in global (Liu and Wang, 2010, 2018). The floodplain has numerous shallow lakes connected with the Yangtze River mainstem (Wang & Dou, 1998). From the 1950s to the 1970s, extensive lateral hydrological connectivity losses occurred in this area (Liu et al., 2018; Wang et al., 2016). Following the loss of lateral hydrological connectivity, the exploitation of lake resources has increased, and the water quality of most disconnected lakes continues to deteriorate, leading to a serious decline in freshwater biodiversity in these areas (Liu et al., 2018; Wang et al., 2016).

A major objective of ecologists is to define the factors that driver biodiversity, mainly in the case of environmental interference, caused by human activities (Butchart et al., 2010; Lindenmayer & Likens, 2010; Simoes et al., 2013). This information knows conservation actions, recommendations of environmental monitoring, and management to reduce the loss of biodiversity (Butchart et al., 2010), and maximizes the use of environmental services through ecosystem maintenance processes (Lindenmayer & Likens, 2010; Millennium Ecosystem Assessment, 2005; Simoes et al., 2013). Knowledge of processes and mechanisms for affecting variations in abundance and biomass of zooplankton is important for maintaining biological balance of freshwater ecosystems (Compte et al., 2016; Waidi et al., 2016). Understanding patterns and mechanisms can explain the community structure in various environments. Information on the species diversity of an ecosystem can better understand ecosystem functioning and biodiversity conservation (Legendre et al., 2005; Magurran, 2011). Here, environmental parameters and community composition of zooplankton, abundance, biomass, species diversity, and diversity of functional taxa in the middle reach of Yangtze River floodplain lakes were monitored from 2019 to 2020. The aims of this study was to (1) analyze the temporal and spatial zooplankton variations and (2) evaluate the environmental parameters affecting zooplankton community structure. We hope our study will provide an important reference for the restoration and protection of freshwater ecosystems in the floodplain lakes.

## 2 MATERIALS AND METHODS

### 2.1 Study area

The study area (28°22′–29°45′N, 115°47′–116°45′E) is located in the southern bank of the Yangtze River in Jiangxi Province, China. Poyang Lake is connected to the Yangtze River, and Junshan Lake, Shanhu Lake, and Qinlan Lake are disconnected to the Yangtze River (Jin et al., 2012). It is a complex and highly interconnected river–lake–wetland system (Jin et al., 2012; Li et al., 2019). It is a humid subtropical climate with significant seasonal changes. The largest amount of precipitation is from April to June (Jin et al., 2012; Li et al., 2019). The study area has a higher water level in the summer and a lower water level in the winter. The average annual temperature and precipitation are 16.3–19.5℃ and 1350–2150 mm, respectively.

**FIGURE 1:**
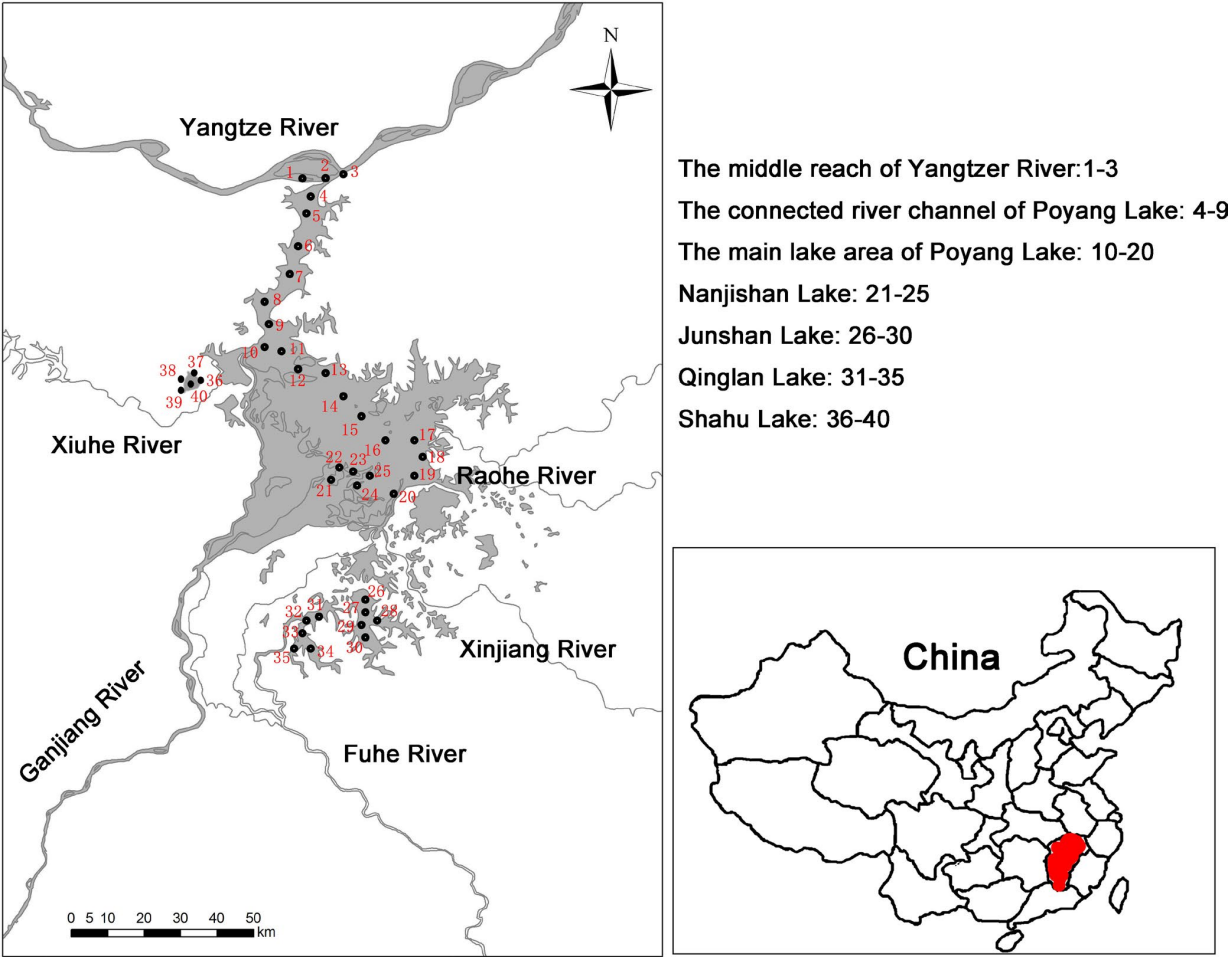
Map of the study area and sampling sites

Selection of sampling sites in this study considered habitat variation and anthropogenic activities in the study area. Samples were collected from seven sampling sections (40 sampling sites; for details, see Figure 1 and Table S1): the middle reach of the Yangtze River (YR; 1–3), the connected river channel of Poyang Lake (TJ; 4–9), the main lake area of Poyang Lake (ML; 10–20), Nanjishan Lake (NJ; 21–25), Junshan Lake (JS; 26–30), Qinglan Lake (QL; 31–35), and Shahu Lake (SH; 36–40).

### 2.2 Sampling methods

Three repeated samples of zooplankton were collected using 25# zooplankton net (64 µm mesh size) in the study areas in April (spring), July (summer), and October (autumn) 2019 and January (winter) 2020. Zooplankton samples for qualitative analysis were collected using 64 µm mesh size and preserved with 10% formaldehyde. A 5 L sampler was used to collect 10 L of mixed surface water samples in six study areas and 25 L of mixed surface water samples in the Yangtze River at approximately 50 cm below the water surface. Zooplankton samples for quantitative analysis were filtered using a 64 µm mesh size, and the sample volume was concentrated to 40 ml. The concentrated sample was preserved with 4% formaldehyde. In the laboratory, the concentrated sample was well stirred for abundance calculations. To count the number of zooplankton, a 5‐ml subsample was observed using a dissecting microscope (Leica S9i) at 10×10 magnification and a microscope (Leica DM500). Biomass of crustaceans was calculated according to the length–weight regression curve, and biomass of Rotifera was calculated according to volume to determine their weight (Nakamura et al., 2017). The zooplankton taxonomic levels were mainly based on the studies of Zhang and Huang (1991), Wang (1961), Jiang and Du (1979), Institute of Zoology and Chinese Academy of Sciences (1979), and Han and Shu (1995).

### 2.3 Measurement of environmental factors

The environmental factors were measured in the study area in April (spring), July (summer), and October (autumn) 2019 and January (winter) 2020. A Multi‐Parameter Probe (YSI, USA) was used to measure the dissolved oxygen (DO; mg/L), hydrogen ions (pH), salinity (Sal; mg/L), turbidity (TURB; NTU), and water temperature (T; °C). A chlorophyll meter (HL‐168C06, made in China) was used to measure the chlorophyll‐*a* (Chl‐*a*; mg/L). A velocity meter (FP111, Global Water, 0.1 m/s accuracy) was used to measure the water velocity (V; m/s). A digital sonar system (H22px handheld sonar system) was used to measure the water depth (WD; m). Water samples were collected and preserved with concentrated H_2_SO_4_, and then refrigerated and transported to the Nanchang University laboratory. Total nitrogen (TN; mg/L) and total phosphorus (TP; mg/L) were analyzed using Ultraviolet Spectrophotometry (Huang et al., 1999; Wei et al., 1989).

### 2.4 Data analysis

Description and classification of zooplankton functional groups were according to Brandl (2005), Barnett et al. (2007), Sun et al. (2010), Benedetti et al. (2015), Shi et al. (2015), Wen et al. (2017), and Ma et al. (2019). The zooplankton community was classified into eight functional groups: rotifers filter feeders (RF), rotifers carnivora (RC), small copepods and cladocerans filter feeders (SCF), small copepods and cladocerans carnivore (SCC), middle copepods and cladocerans filter feeders (MCF), middle copepods and cladocerans carnivore (MCC), large copepods and cladocerans filter feeders (LCF), and large copepods and cladocerans carnivore (LCC).

To analyze the species diversity and richness of zooplankton in each sampling section, four biodiversity indices (Shannon–Wiener index: *H*′; Simpson index: *C*; Pielou evenness index: *J*′; Margalef index: *R*) were assessed (Magurran, 1988; Peet, 1974). To analyze the difference in species composition between different communities, we used the beta diversity decomposition method (BAS frameworks; Baselga, 2010; Carvalho et al., 2012) based on the Sørensen index (β_sor_), species spatial turnover (β_sim_), and nestedness components (β_sne_).

To explore the potential mechanisms of changes in species diversity, abundance, and biomass, the correlations between the species diversity, abundance, biomass, and environmental factors were analyzed using Mantel tests with 9999 permutations (Legendre & Legendre, 2012). β diversity analyses and Mantel tests were performed in R 3.2.0 (R Development Core Team, 2014) using the BETAPART package (Baselga & Orme, 2012) and VEGAN (Oksanen et al., 2015).

One‐way analysis of variance (ANOVA) was used to test significant variance (*p < .05; **p < .01; ***p < .001) between species diversity, abundance, biomass, and environmental factors among each section and each season. The SPSS 22.0 was used to perform ANOVA tests.

The nonmetric multidimensional scaling (NMDS) ordination plots were used to assess the variation in the composition of the zooplankton community among sampling sections. The ordination plots were based on Bray–Curtis index of the zooplankton taxa from sampling sections. The nonmetric multidimensional scaling ordination plots and Bray–Curtis index were performed using PRIMER 6 (Clarke & Gorley, 2006).

We analyzed the effect of environmental factors on the composition of zooplankton community using redundancy analysis (RDA; ter Braak & Verdonschot, 1995; Lep & Smilauer, 2003). To determine whether linear or unimodal ordination, we performed a detrended correspondence analysis (DCA) for the composition of zooplankton community (Lep & Smilauer, 2003). Monte Carlo permutation tests with 999 permutations were used to test significant variance (*p *< .05) of the RDA gradient (ter Braak & Verdonschot, 1995). The environmental factors and the composition of zooplankton community were log10(*X* + 1) transformed to meet assumptions of multivariate normality and moderate the influence of extreme data (Borcard et al., 2011). Redundancy analysis was performed CANOCO Version 4.5 (ter Braak & Verdonschot, 1995).

## 3 RESULTS

### 3.1 Taxonomic composition of zooplankton

**FIGURE 2:**
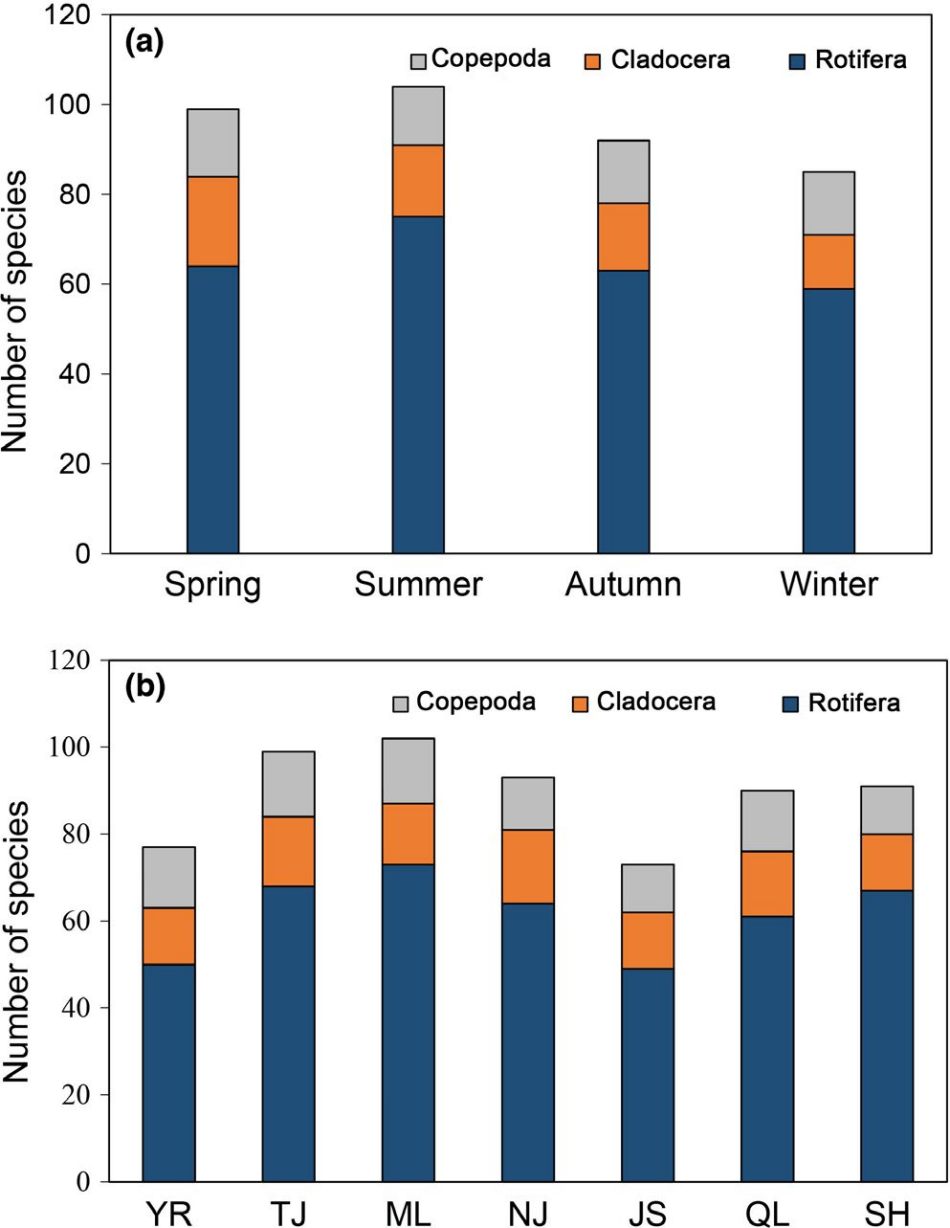
Seasonal (a) and spatial (b) variations in the number of zooplankton species in the Yangtze River floodplain lakes. YR: the middle reach of the Yangtze River; TJ: the connected river channel of Poyang Lake; ML: the main lake area of Poyang Lake; NJ: Nanjishan Lake; JS: Junshan Lake; QL: Qinglan Lake; SH: Shahu Lake

Zooplankton samples from the middle reach of Yangtze River floodplain lakes were classified into 128 species, 61 genus, and 26 families (Table S2). Rotifera was the most common taxon, accounting for 68.8% (88) in the total number of species (Figure 2). Significant seasonal differences were detected among the number of zooplankton species (ANOVA, *F_df_*_1,_ *_df_*_2_ = 4.401, *p* = .013), but no significant spatial differences (ANOVA, *F_df_*_1,_ *_df_*_2_ = 2.178, *p* = .086). The number of species in summer (104) was greater than it was in other seasons (Figure 2a). The ML had the highest number of species, followed by the TJ, and the number of species in YR was the lowest (Figure 2b).

### 3.2 Quantitative assessment of zooplankton

**FIGURE 3:**
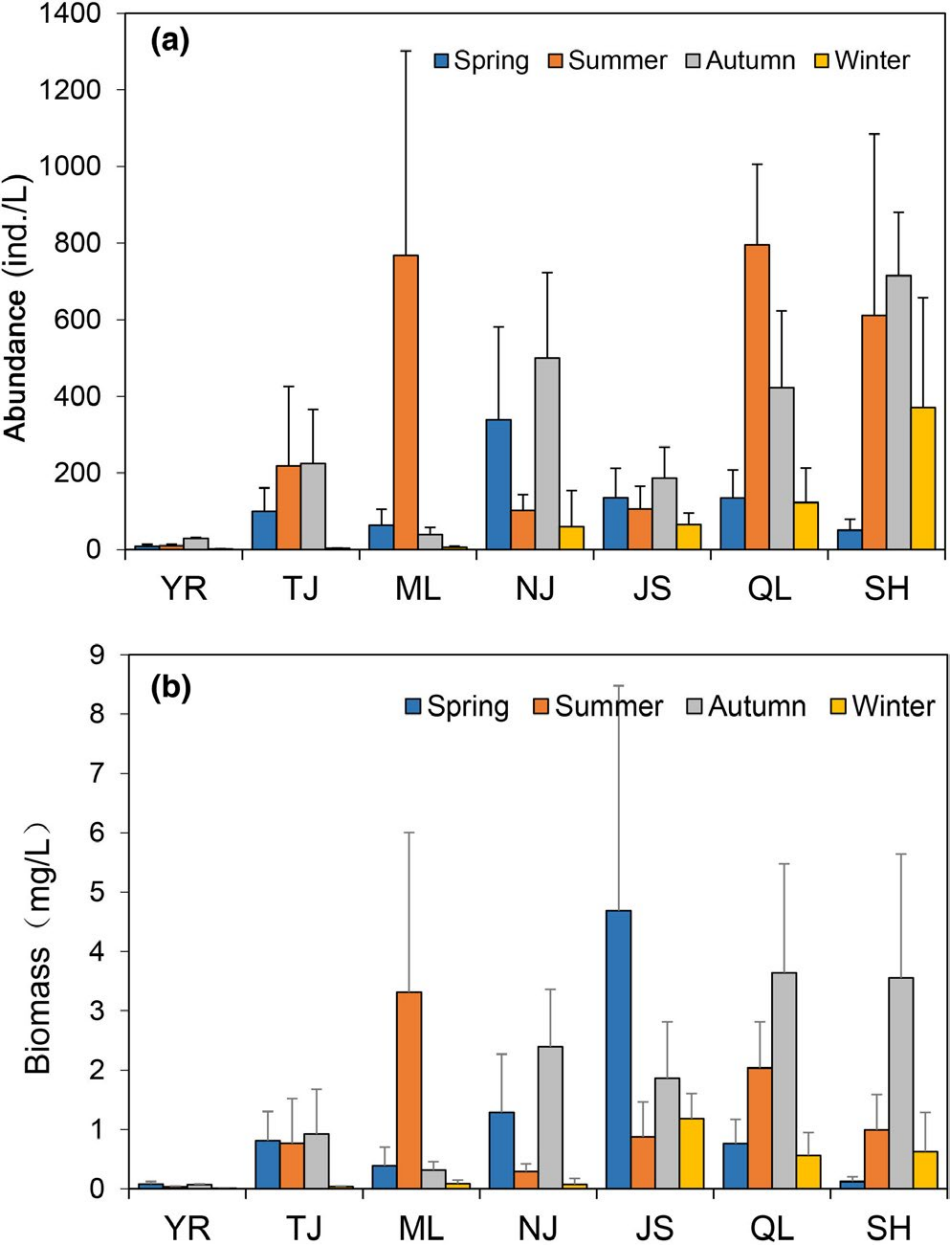
Seasonal variations in the abundance and biomass of zooplankton in the Yangtze River floodplain lakes. YR: the middle reach of the Yangtze River; TJ: the connected river channel of Poyang Lake; ML: the main lake area of Poyang Lake; NJ: Nanjishan Lake; JS: Junshan Lake; QL: Qinglan Lake; SH: Shahu Lake

**FIGURE 4:**
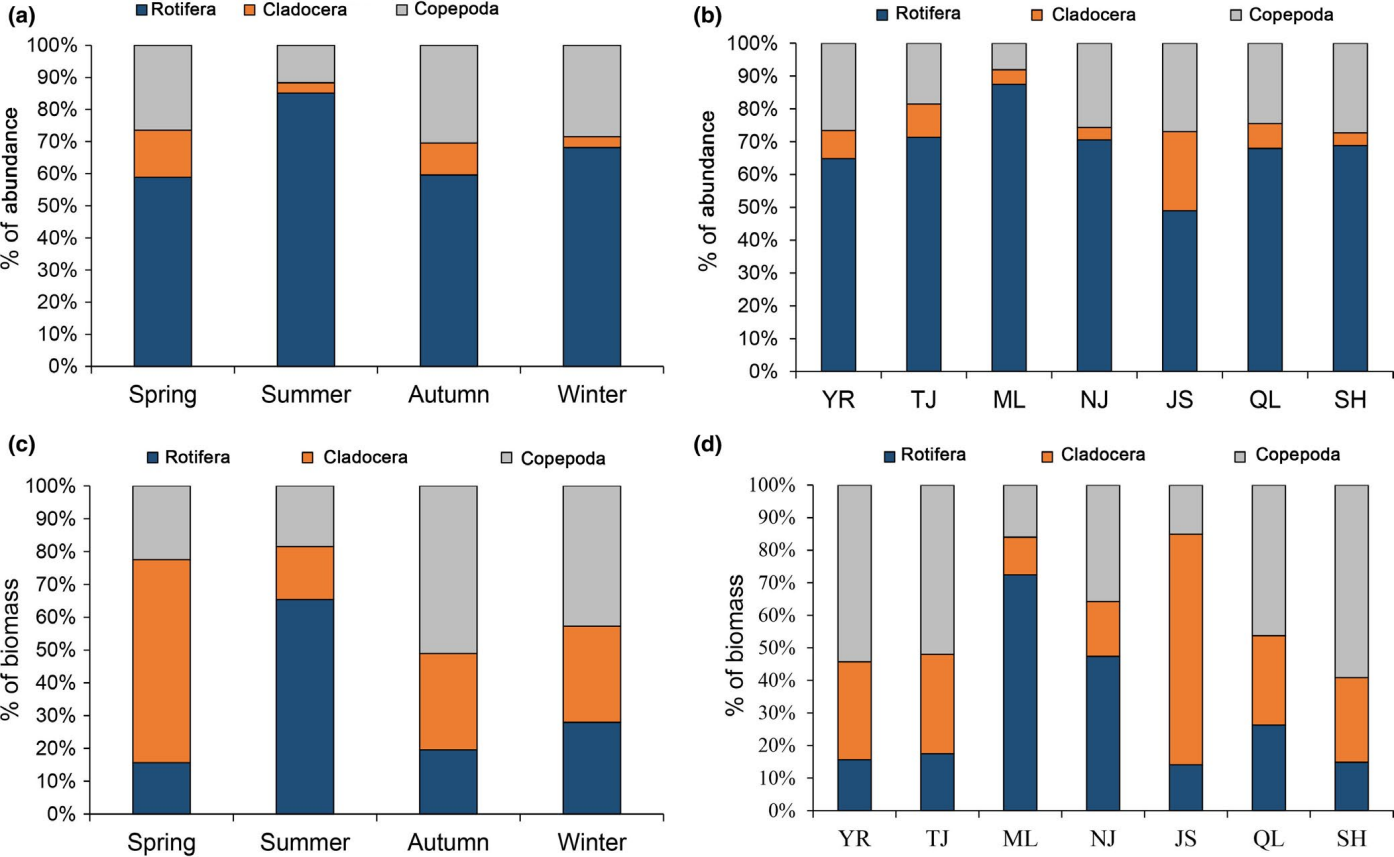
Seasonal (a, c) and spatial (b, d) variations in the abundance and biomass of zooplankton taxa in the Yangtze River floodplain lakes. YR: the middle reach of the Yangtze River; TJ: the connected river channel of Poyang Lake; ML: the main lake area of Poyang Lake; NJ: Nanjishan Lake; JS: Junshan Lake; QL: Qinglan Lake; SH: Shahu Lake

The mean abundance and biomass of zooplankton in the middle reach of Yangtze River floodplain lakes were 221.1 ind/L and 1.14 mg/L, respectively. Significant seasonal differences were found among the abundance and biomass of zooplankton (abundance: ANOVA, *F_df_*_1,_ *_df_*_2_ = 13.122, *p* < .001; biomass: ANOVA, *F_df_*_1,_ *_df_*_2_ = 5.162, *p* = .002), but no significant spatial differences were found (abundance: ANOVA, *F_df_*_1,_ *_df_*_2_ = 1.141, *p* = .289; biomass: ANOVA, *F_df_*_1,_ *_df_*_2_ = 0.912, *p* = .619). The abundance and biomass in summer and autumn were higher than those that in other seasons (Figure 3a). The abundance in NJ and SH was higher than those that in other sampling sections (Figure 3b), and the biomass in JS and NJ was higher (Figure 3b). Similarly, we found significant seasonal and spatial differences among the abundance of taxonomic groups (seasonal differences: ANOVA, *F_df_*_1,_ *_df_*_2_ = 10.497, *p* < .001; spatial differences: ANOVA, *F_df_*_1,_ *_df_*_2_ = 4.594, *p* < .001), but no significant seasonal and spatial differences were found among the biomass of taxonomic groups (seasonal differences: ANOVA, *F_df_*_1,_ *_df_*_2_ = 0.118, *p* = .888; spatial differences: ANOVA, *F_df_*_1,_ *_df_*_2_ = 1.250, *p* = .271). The abundance of Rotifera in the seasonal and spatial changes was higher than those that in other taxa (Figure 4a,b). The biomass of Cladocera in spring and JS was higher than those that in other taxa (Figure 4c). The biomass of Rotifera in summer, and the biomass of ML and NJ was higher than those that in other taxa (Figure 4c,d). The biomass of Copepoda in autumn and winter, and the biomass of YR, TJ, QL, and SH was higher than those that in other taxa (Figure 4c,d).

### 3.3 Composition and biomass of zooplankton functional groups

**FIGURE 5:**
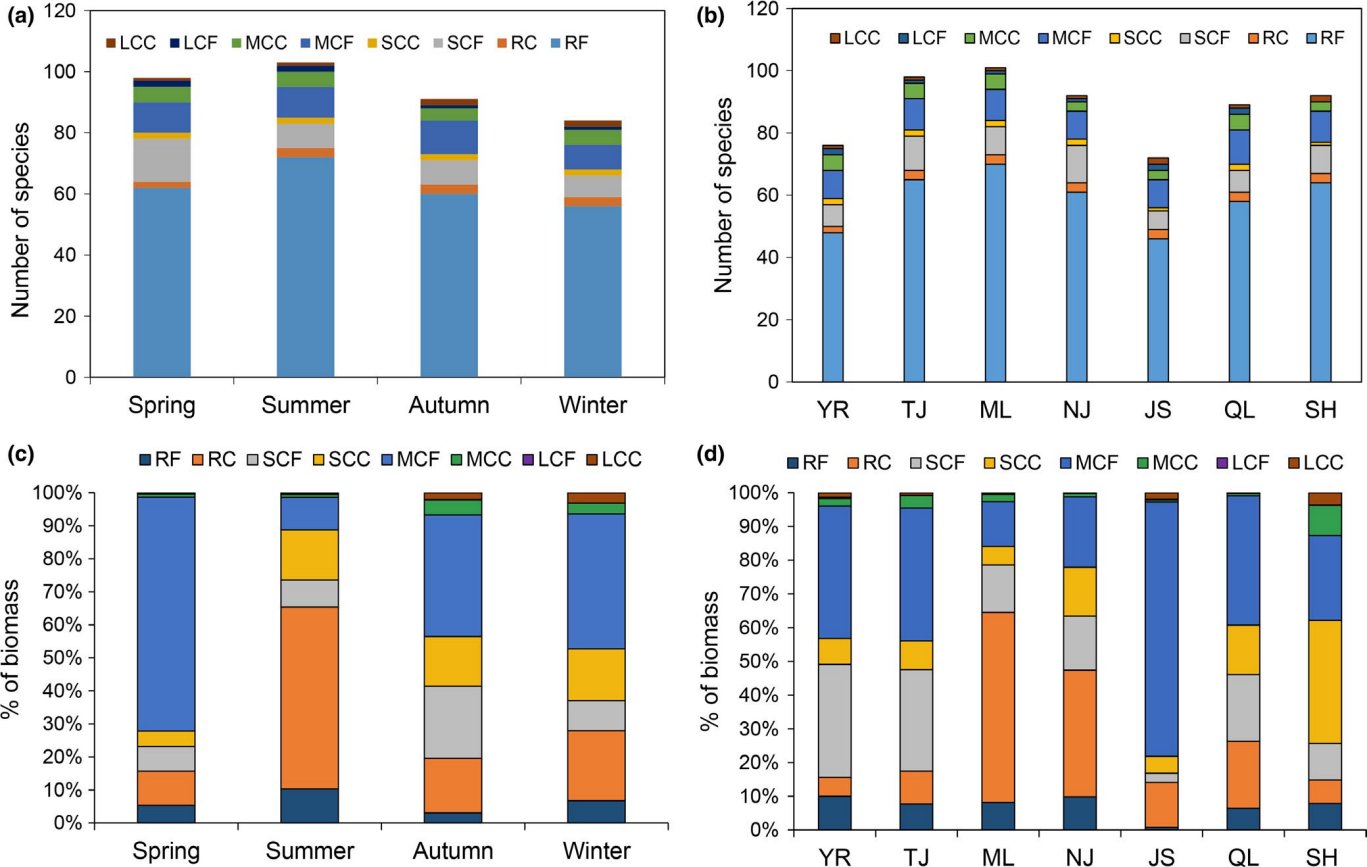
Seasonal (a, c) and spatial (b, d) variations in the abundance and biomass of zooplankton functional groups in the Yangtze River floodplain lakes. YR: the middle reach of the Yangtze River; TJ: the connected river channel of Poyang Lake; ML: the main lake area of Poyang Lake; NJ: Nanjishan Lake; JS: Junshan Lake; QL: Qinglan Lake; SH: Shahu Lake

No significant seasonal and spatial differences were found among the species number of zooplankton functional groups (seasonal differences: ANOVA, *F_df_*_1,_ *_df_*_2_ = 0.037, *p* = .999; spatial differences: ANOVA, *F_df_*_1,_ *_df_*_2_ = 0.020, *p* = .996). The number of RF species was the highest, followed by MCF and SCF, and the number of species in LCC and LCF was the lowest (Figure 5a,b). Similarly, significant seasonal differences were found among the biomass of zooplankton functional groups (ANOVA, *F_df_*_1,_ *_df_*_2_ = 4.054, *p* < .001), but no spatial differences were found (ANOVA, *F_df_*_1,_ *_df_*_2_ = 1.203, *p* = .234). In the seasonal changes, the biomass of MCF in spring, autumn, and winter and the biomass of RC in summer were higher than those that in other functional groups (Figure 5c,d). In the spatial changes, the biomass of MCF in YR, TJ, JS, and QL and the biomass of RC in ML and NJ were higher than those that in other functional groups (Figure 5c,d).

### 3.4 Diversity patterns of zooplankton

**FIGURE 6:**
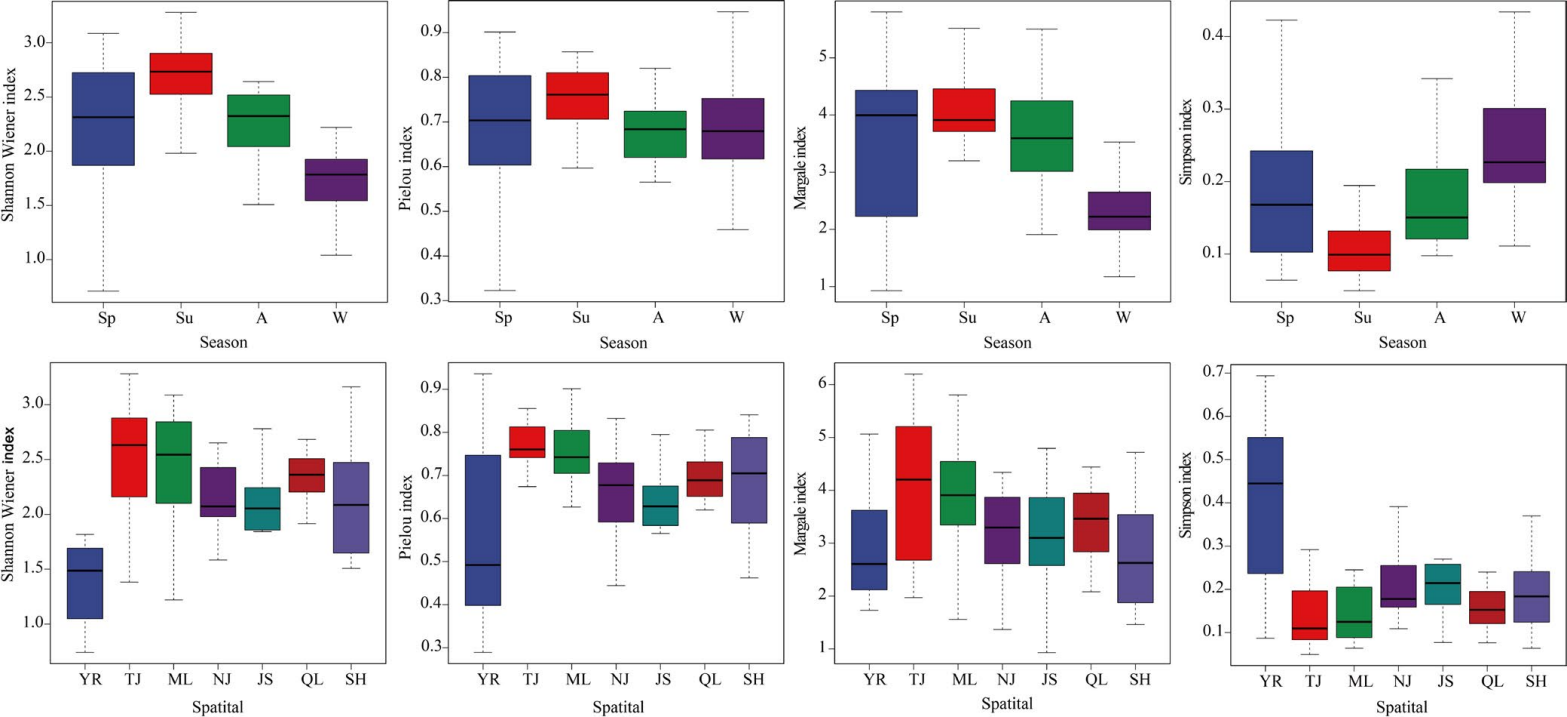
Seasonal and spatial variations in the α diversity of zooplankton in the Yangtze River floodplain lakes. The horizontal lines represent median, and the vertical lines represent range. YR: the middle reach of the Yangtze River; TJ: the connected river channel of Poyang Lake; ML: the main lake area of Poyang Lake; NJ: Nanjishan Lake; JS: Junshan Lake; QL: Qinglan Lake; SH: Shahu Lake

Significant seasonal and spatial differences were found among the diversity of zooplankton (seasonal differences: ANOVA, *F_df_*_1,_ *_df_*_2_ = 2.832, *p* = .049; spatial differences: ANOVA, *F_df_*_1,_ *_df_*_2_ = 2.911, *p* = .032). The highest diversity was found in summer, followed by autumn, and the diversity in winter was the lowest (Figure 6). In the spatial changes, the highest diversity was found in TJ, followed by ML and QL, and the diversity in YR was the lowest (Figure 6).

**FIGURE 7:**
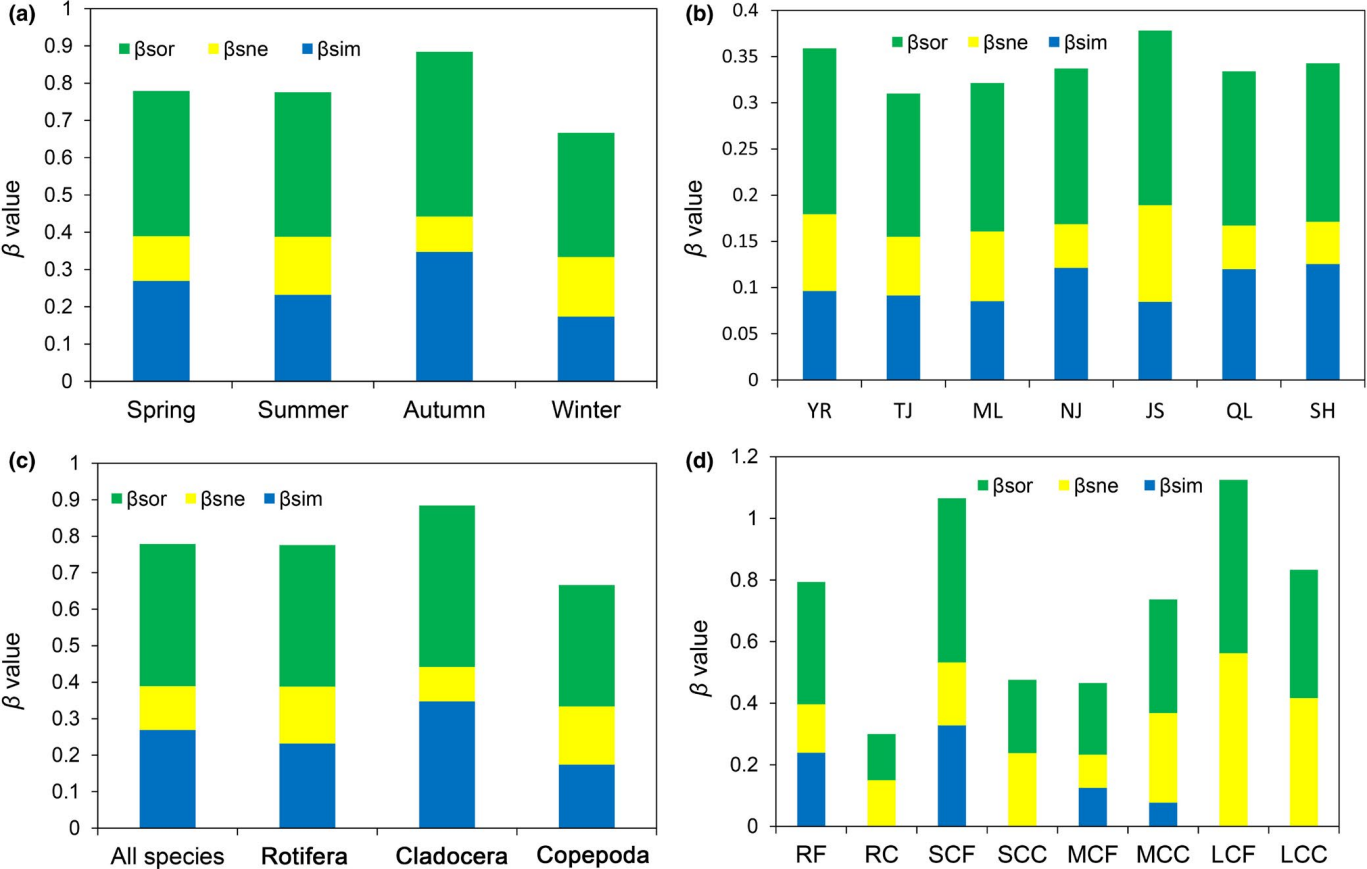
Seasonal (a) and spatial (b) variations in the β diversity of zooplankton, and the β diversity of taxonomic (c) and functional groups (d) in the Yangtze River floodplain lakes. YR: the middle reach of the Yangtze River; TJ: the connected river channel of Poyang Lake; ML: the main lake area of Poyang Lake; NJ: Nanjishan Lake; JS: Junshan Lake; QL: Qinglan Lake; SH: Shahu Lake. Sp: spring; Su: summer; A: autumn; W: winter

The composition dissimilarity of zooplankton had a lower value of 0.39. In the seasonal changes, the composition dissimilarity of zooplankton in the Yangtze River floodplain lakes in autumn (0.44) was higher than it was in other seasons (Figure 7a). In the spatial changes, the highest composition dissimilarity was found in JS (0.19), followed by YR (0.18), and the composition dissimilarity in TJ (0.16) was the lowest (Figure 7b). In the composition dissimilarity changes of taxonomic groups, the highest composition dissimilarity was found in Cladocera (0.44), followed by Rotifera (0.39), and the composition dissimilarity in Copepoda (0.33) was the lowest (Figure 7c). The spatial turnover component was greater than the nestedness component in each season, each section, and each taxonomic group (Figure 7). In the composition dissimilarity changes of functional groups, the highest composition dissimilarity was found in LCF (0.56), followed by SCF (0.53), and the composition dissimilarity in RC (0.15) was the lowest (Figure 7d). The spatial turnover component was greater than the nestedness component in RF, SCF, and MCF, but the nestedness component in MCC was the main contributor to the beta diversity (Figure 7d). In addition, the β_sim_ was zero in RC, SCC, LCF, and LCC, which indicate that the nestedness component was the entire contributor to the β diversity among them (Figure 7d).

### 3.5 Temporal and spatial variation in zooplankton community

**FIGURE 8:**
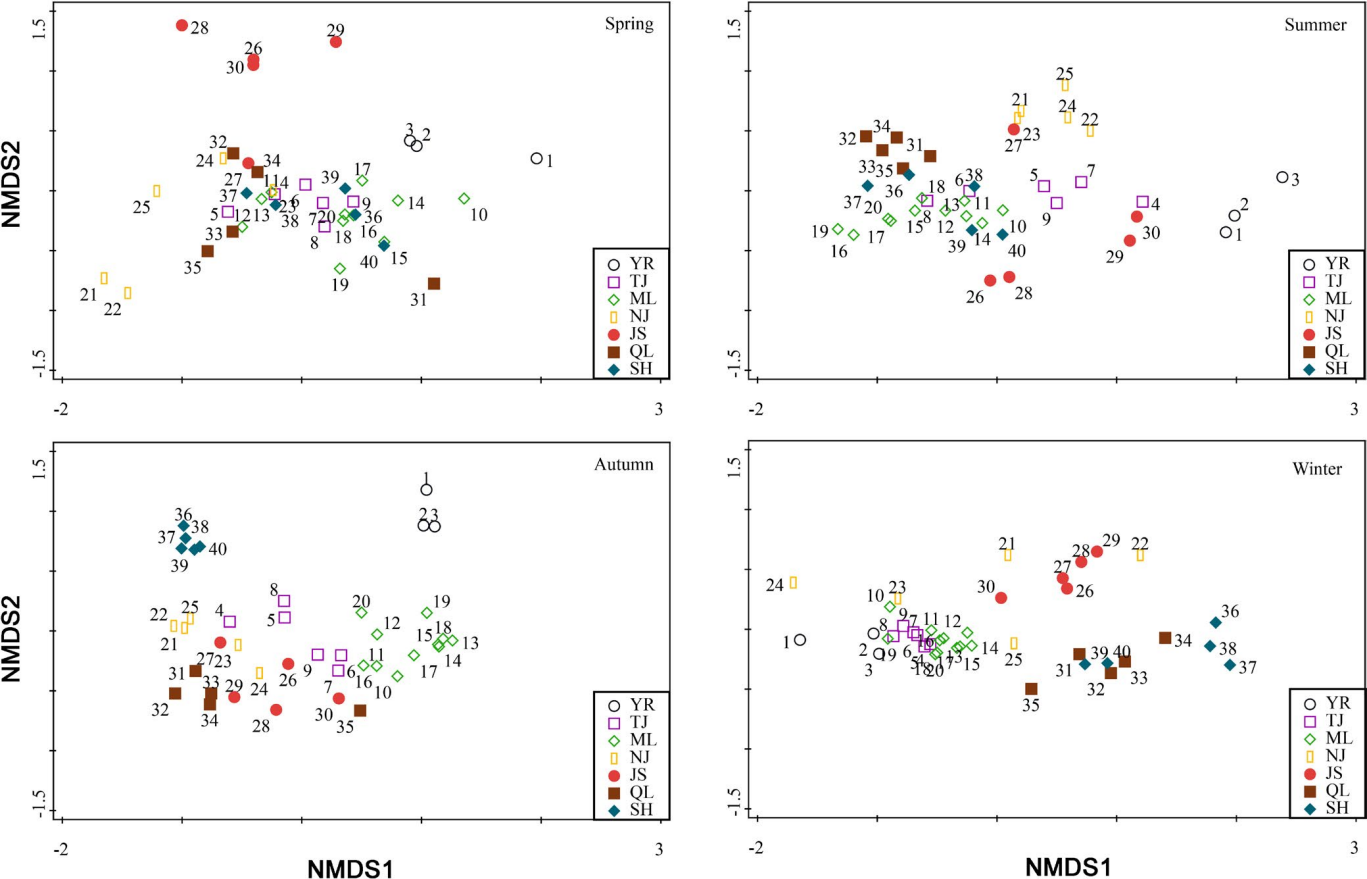
The nonmetric multidimensional scaling (NMDS) ordination in the structure zooplankton community in the Yangtze River floodplain lakes. YR: the middle reach of the Yangtze River; TJ: the connected river channel of Poyang Lake; ML: the main lake area of Poyang Lake; NJ: Nanjishan Lake; JS: Junshan Lake; QL: Qinglan Lake; SH: Shahu Lake

**FIGURE 9:**
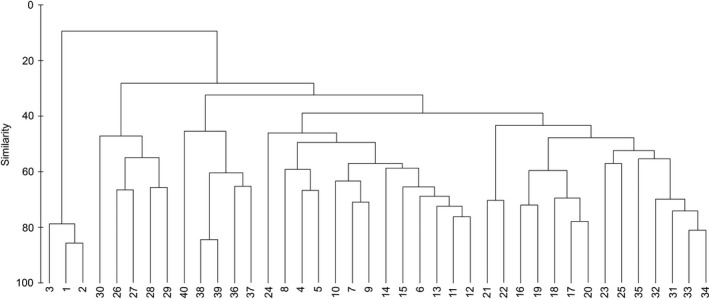
The Bray–Curtis resemblance matrix in the structure zooplankton community in total year from the middle reach of Yangtze River floodplain lakes. 1–40: Sampling sites, see Figure 1

The NMDS plot showed that the structure of zooplankton community exhibited significant seasonal changes. In spring, YR and JR formed one cluster, respectively, and the structure of zooplankton community in other sampling sections was similar (Figure 8 and Figure S1). In summer, YR and NJ formed one cluster, respectively, and the structure of zooplankton community in other sampling sections was similar (Figure 8 and Figure S1). In autumn, YR and SH formed one cluster, respectively, and the structure of zooplankton community in other sampling sections was similar (Figure 8 and Figure S1). In winter, JS formed first cluster, SH and QL formed second cluster, and the structure of zooplankton community in other sampling sections formed third cluster (Figure 8 and Figure S1). The Bray–Curtis similarity showed that the structure of zooplankton community in total year from the middle reach of Yangtze River floodplain lakes was divided into five areas, in which the first area included TJ and ML; the second area included NJ, QL, and ML; and YR, JS and SH formed one cluster, respectively (Figure 9).

### 3.6 Association between the structure of zooplankton community and environmental factors

**FIGURE 10:**
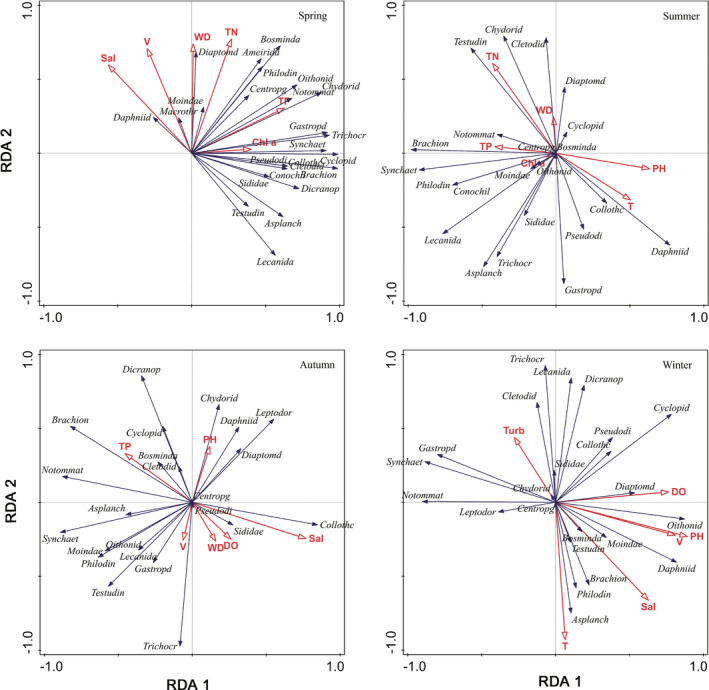
Ordination biplot of major zooplankton species and environmental variables based on redundancy analysis in the Yangtze River floodplain lakes. DO: dissolved oxygen (mg/L); pH: hydrogen ions; Sal: salinity (mg/L); TURB: turbidity (NTU); T: water temperature (°C); Chl‐*a*: chlorophyll‐*a* (mg/L); V: water velocity (m/s); WD: water depth (m); TN: total nitrogen (mg/L); TP: total phosphorus (mg/L)

Redundancy analysis (RDA) showed eigenvalues along the first axis were 0.781, 0.461, 0.395, and 0.412 in spring, summer, autumn, and winter, respectively. The cumulative percentage variance of the species–environment relation along the first axis was 78.1%, 46.1%, 39.5%, and 41.2% in spring, summer, autumn, and winter, respectively. The structure of zooplankton community in spring was correlated with chlorophyll‐*a* and total phosphorus (Figure 10). The structure of zooplankton community in summer showed a correlation with pH, water depth, and total nitrogen (Figure 10). The structure of zooplankton community in autumn presented a correlation with salinity and pH (Figure 10). Additionally, the structure of zooplankton community in winter had a correlation with pH and water temperature (Figure 10). In addition, water depth and water velocity was correlated with abundance, biomass, and α diversity, and salinity was significantly associated with α diversity and β diversity of zooplankton, based on the Mantel test (*p* < .05; Table 1).

**TABLE 1:** Effects of physicochemical parameter on pairwise abundance, biomass, α diversity, and β diversity in the Yangtze River floodplain lakes

		*B*	*D*	α diversity				β diversity		
				*H*	*J*	*R*	*C*	β_sor_	β_sim_	β_sne_
T	*r*	.15	.01	−.32	−.33	−.36*	−.24	.10	.35	−.19
	*p*	.29	.42	.06	.12	.**04**	.23	.22	.19	.33
TURB	*r*	−.30	.02	−.24	−.29	−.30	−.18	.21	.31	−.10
	*p*	.07	.40	.18	.14	.11	.30	.13	.21	.38
Sal	*r*	.51	.41	.91*	.79**	.21	.96**	.49*	−.38	.55*
	*p*	.10	.11	.**03**	.**01**	.19	.**01**	.**05**	.09	.**04**
DO	*r*	−.17	−.03	−.18	−.22	−.12	−.13	.01	.38*	−.26*
	*p*	.30	.53	.17	.13	.39	.47	.29	.**02**	.**02**
V	*r*	.50*	.34*	.58**	.56*	−.05	.59*	−.03	.10	−.02
	*p*	.**03**	.**05**	.**01**	.**05**	.59	.**05**	.46	.48	.53
Chl‐*a*	*r*	.19	.29	.19	.10	−.26	.27	.31	.12	.09
	*p*	.23	.14	.24	.28	.25	.10	.15	.49	.34
pH	*r*	.37	−.22	.02	.11	−.20	−.08	−.02	−.22	.14
	*p*	.08	.21	.55	.41	.23	.57	.52	.11	.28
TP	*r*	.23	.04	−.13	−.17	−.35*	−.10	.49	.04	.25
	*p*	.12	.46	.43	.29	.**03**	.66	.07	.45	.13
TN	*r*	.38	−.22	−.08	−.11	−.21	−.07	.40	−.34	.47
	*p*	.23	.24	.58	.54	.38	.74	.20	.10	.15
WD	*r*	.49*	.53*	.70*	.64*	−.03	.74*	.08	−.01	.05
	*p*	.**05**	.**04**	.**05**	.**04**	.56	.**03**	.37	.46	.34

Note

Significant results are in bold (*p < .05; **p < .01).

Abbreviations: *B*, biomass (mg/L); *C*, Simpson index; Chl‐*a*, chlorophyll‐*a* (mg/L); *D*, abundance (ind/L); DO, dissolved oxygen (mg/L); *H*, Shannon–Wiener index; *J*, Pielou evenness index; pH, hydrogen ions; *R*, Margalef index; Sal, salinity (mg/L); T, water temperature (°C); TN, Total nitrogen (mg/L); TP, total phosphorus (mg/L); TURB, turbidity (NTU); V, water velocity (m/s); WD, water depth (m); β_sim_, spatial turnover component; β_sne_, nestedness component; β_sor_, compositional dissimilarity.

## 4 DISCUSSION

### 4.1 Temporal and spatial variations in zooplankton diversity

The Yangtze River Basin is one of the most human‐influenced drainage basins worldwide (Liu & Diamond, 2005; Yang et al., 2011; Zhang et al., 2020), and fisheries’ sustainability and biodiversity conservation in this basin have both faced great challenges during the past 70 years (Liu et al., 2019; Wu et al., 2004; Xie et al., 2003; Zhang et al., 2020). Anthropogenic activities have adversely affected the Yangtze River's aquatic organisms and their habitats with continual socioeconomic development (Lu et al., 2016; Zhang et al., 2017, 2020). In this study, to determine the seasonal and spatial zooplankton variations and association of water quality, the diversity and structure of zooplankton community were analyzed in the middle reach of Yangtze River floodplain lakes. The results showed that significant seasonal and spatial differences were found among the diversity and community composition of zooplankton. In addition, this study also showed that environmental factors are important for affecting the structure of zooplankton community.

There was a significant seasonal variation in α diversity and community composition of zooplankton. The species number, abundance, biomass, and α diversity of zooplankton were the highest in summer rather than during the other sampling periods. This may be attributed to the favorable summer temperatures, and phytoplankton was abundant in summer for increasing the number of zooplankton (Liu et al., 2020; Qin et al., 2020). In addition, the floodplain lake habitats became more complex in different seasons due to the presence of different morphological types of aquatic macrophytes (emergent, floating, rooted with floating leaves and submerged; Liu, 2016; Thomaz et al., 2009). The diverse aquatic macrophytes could increase not only local, but also regional biodiversity (Lemmens et al., 2013), and played a major role in shaping the zooplankton communities (Bolduc et al., 2016; Nhiwatiwa et al., 2017). β diversity of zooplankton declined from spring to summer, increased in autumn, and decreased in winter with the lowest value. It indicated that zooplankton communities tended to homogenize in summer, which may be attributed to the flooding period increased connectivity and water area of lake habitats, resulting in a decrease of β diversity of zooplankton in species turnover (Alves et al., 2005; Gonzalez, 2009; Simões et al., 2013).

We also found significant spatial variation in α diversity and community composition of zooplankton. The species number, abundance, biomass, and α diversity of zooplankton in ML and TJ were higher than those that in other sampling sections, which may be attributed to water area of lake habitats. Some studies also showed that the abundance of Crustacea is usually positively correlated with lake area because habitat diversity usually increases with area (Gooriah & Chase, 2020; Hoffmann & Dodson, 2005; Stanley, 1992). The concentrations of nutrients of ML and TJ may be higher than those that in the other areas. The higher concentrations of nutrients increased in Cladocera and Copepoda abundance with a concurrent (DeBoer et al., 2015; Pace, 1986). The species number, abundance, biomass, and α diversity of zooplankton in JS were lower than those that in other lakes, which may be related to lower primary productivity and higher predation pressure (Dodson et al., 2000; Leibold, 1999; Stanley, 1992; Waide et al., 1999). The ratio of macrophytes and phytoplankton is important for maintaining a favorable transparency in lakes (Scheffer, 1998; Zhang et al., 2016). Shallow lakes often exhibit alternative states with transparent water occupied by macrophytes, while turbid water is occupied by phytoplankton (Scheffer, 1998; Zhang et al., 2016). The diversity of zooplankton may vary with fish in different habitats (Achenbach & Lampert, 1997; Gliwicz & Pijanowska, 1989). The main fish of enclosure aquaculture in JS were four domestic Chinese carp species, and the pressure on zooplankton was preyed by fish increased. The species number, abundance, biomass, and α diversity of zooplankton in YR were the lowest, which may be attributed to the rapid water flow of YR is not good for the growth and survival of zooplankton (Thorp & Mantovani, 2005).

### 4.2 Temporal and spatial variations in zooplankton functional groups

Functional traits consider morphological, physiological, ecological, and behavioral characteristics of species, which can determine the response of species to ecosystem processes and environmental characteristics (Braghin et al., 2018; Violle et al., 2007). In addition, functional traits detect processes that drive communities’ changes, and evaluate and monitor environmental impacts (Arrieira et al., 2015; Braghin et al., 2018; Laureto et al., 2015; Oliveira et al., 2018). Zooplankton have different functional traits in floodplains, where its distribution is influenced by environmental filters, such as concentration of nutrient and chlorophyll‐*a*, water depth, and transparency (Bozelli et al., 2015; Chaparro et al., 2011; Lansac‐Tôha et al., 2009; Simões et al., 2013). We found significant temporal and spatial variations in the species number and biomass of zooplankton functional groups. The biomass of rotifers carnivore (RC) in the main lake area of Poyang Lake and Nanji Lake was higher than those that in other functional groups, which may be attributed to they have abundant macrophyte and higher concentration of nutrient. Some studies showed that zooplankton like habitat in macrophyte beds because they may be affect the ratio of functional groups based on creating microhabitats and altering the top‐down and bottom‐up interactions (Bolduc et al., 2016; Heino, 2008; Kuczyńska‐Kippen, 2005; Thomaz & Cunha, 2010; Viana et al., 2016). In addition, the biomass of small copepods and cladocerans filter feeders (SCF) and middle copepods and cladocerans filter feeders (MCF) in the middle reach of the Yangtze River and the connected river channel of Poyang Lake was higher than those that in other functional groups, which may be attributed to the relatively rapid water flow. A higher water flow could reduce the abundance of Rotifera, while Crustacea are larger and could adapt to this environmental characteristic (Thorp & Mantovani, 2005).

### 4.3 Key environmental factors for driving structure of zooplankton community

The temporal and spatial differences in the structure of zooplankton community may be affected by many environmental factors, such as water temperature, water depth, chlorophyll‐*a*, total phosphorus, total nitrogen, and pH in the floodplain lakes (Du et al., 2014; Lacerot et al., 2013; Miron et al., 2014; Okogwu, 2009; Wang et al., 2017). The structure of zooplankton community in this study was significantly associated with chlorophyll‐*a*, salinity, total phosphorus, water temperature, water depth, pH, and total nitrogen. Water temperature is important environmental factor for affecting structure of zooplankton community (Jiang et al., 2015; Kagalou et al., 2010). For example, Helland et al. (2007) found that water temperature was the key environmental factor for determining structure of zooplankton community in Lake Stechlin of Germany. The increase in concentration of nutrient could promote the growth of phytoplankton, and indirectly affect the structure of zooplankton community based on the effect of top‐down interactions from phytoplankton (Wu et al., 2011). In spring and summer, chlorophyll‐*a* and nutrients were the main environmental factors affecting the diversity of zooplankton in this study. Additionally, some studies also showed that zooplankton taxonomic richness was significantly associated with pH (Shurin et al., 2009). Our study showed that pH was negatively correlated with the structure of zooplankton community.

### 4.4 Linking beta diversity patterns to habitat restoration and its driver factors

β diversity can determine the number of habitat restoration areas needed (Anderson et al., 2006; Margules & Pressey, 2000; Wiersma & Urban, 2005). If the spatial turnover component is the mainly contributor to β diversity, a larger number of habitat restoration areas would be necessary to restore regional biodiversity (Baiser et al., 2012; Carvalho et al., 2012). On the contrary, the nestedness component is the main contributor to β diversity, and habitat restoration areas with a low species richness would be necessary to restore regional biodiversity (Baiser et al., 2012; Carvalho et al., 2012). This study showed that the spatial turnover component in the middle reach of Yangtze River floodplain lakes was the main contributor to the β diversity, which indicated that all study areas should establish habitat restoration areas to restore regional biodiversity.

The driver factors of spatial turnover component include habitat loss, geographical isolation, dispersal restrictions, environmental filter, and competition (Angeler, 2013; Gutiérrez‐Cánovas et al., 2013; Legendre, 2014), while nestedness component includes selective extinction, selective colonization, passive sampling, and habitat nestedness (Baselga, 2010; González‐Oreja et al., 2012; Wang et al., 2012). This study showed that the spatial turnover component in the middle reach of Yangtze River floodplain lakes was the main contributor to the β diversity for each taxonomic group, which indicated that habitat loss, geographical isolation, dispersal restrictions, environmental filter, and competition may be the main driver factors. For analysis of functional groups, the spatial turnover component was the main contributor to the β diversity in rotifers filter feeders (RF), SCF, and MCF, while the nestedness component in middle copepods and cladocerans carnivore (MCC) was the main contributor. In addition, the nestedness component was the entire contributor to the β diversity in RC, small copepods and cladocerans carnivore (SCC), large copepods and cladocerans filter feeders (LCF), and large copepods and cladocerans carnivore (LCC), which indicates that selective extinction may be the main driver factors.

## 5 CONCLUSION

This study proved that the seasonal and spatial changes in the structure of zooplankton community respond to changes in environmental factors in the middle reach of Yangtze River floodplain lakes. In addition, variations of habitat characteristics and seasonal change were also the main driver factors for the variations of zooplankton communities. These results indicated that zooplankton species was increasingly vulnerable, and they could constitute a useful indicator for monitoring water quality. Additionally, anthropogenic activity posed a great threat to the freshwater ecosystem based on beta diversity pattern. Thus, more attention should be focused on anthropogenic habitat alteration in the Yangtze River floodplain lakes.

## CONFLICT OF INTEREST

None declared.

## AUTHOR CONTRIBUTION

**Quanfeng Lu:** Conceptualization (equal); Data curation (equal); Formal analysis (equal); Investigation (equal); Methodology (equal); Resources (equal); Software (equal); Writing‐original draft (equal); Writing‐review & editing (equal). **Xiongjun Liu:** Conceptualization (equal); Data curation (equal); Formal analysis (equal); Investigation (equal); Methodology (equal); Software (equal); Writing‐original draft (equal); Writing‐review & editing (equal). **Xuemei Qiu:** Investigation (equal); Methodology (equal). **Tao Liang:** Investigation (equal); Resources (equal). **Jinping Chen:** Investigation (equal). **Shuai Zhao:** Investigation (equal). **Shan Ouyang:** Writing‐original draft (equal); Writing‐review & editing (equal). **Binsong Jin:** Writing‐original draft (equal); Writing‐review & editing (equal). **Xiaoping Wu:** Funding acquisition (equal); Project administration (equal); Validation (equal); Visualization (equal); Writing‐original draft (equal); Writing‐review & editing (equal).

## Supporting information

Fig S1

Table S1

Table S2

## Data Availability

The data used in this study are archived in the Dryad Data Repository (https://doi.org/10.5061/dryad.5qfttdz5h).
