# Supplementary figures and images for: Changes and drivers of zooplankton diversity patterns in the middle reach of Yangtze River floodplain lakes, China
Source: Ecol Evol. 2021 Dec 15;11(24):17885–900. doi: 10.1002/ece3.8353 (PMC8717274; doi:10.1002/ece3.8353)

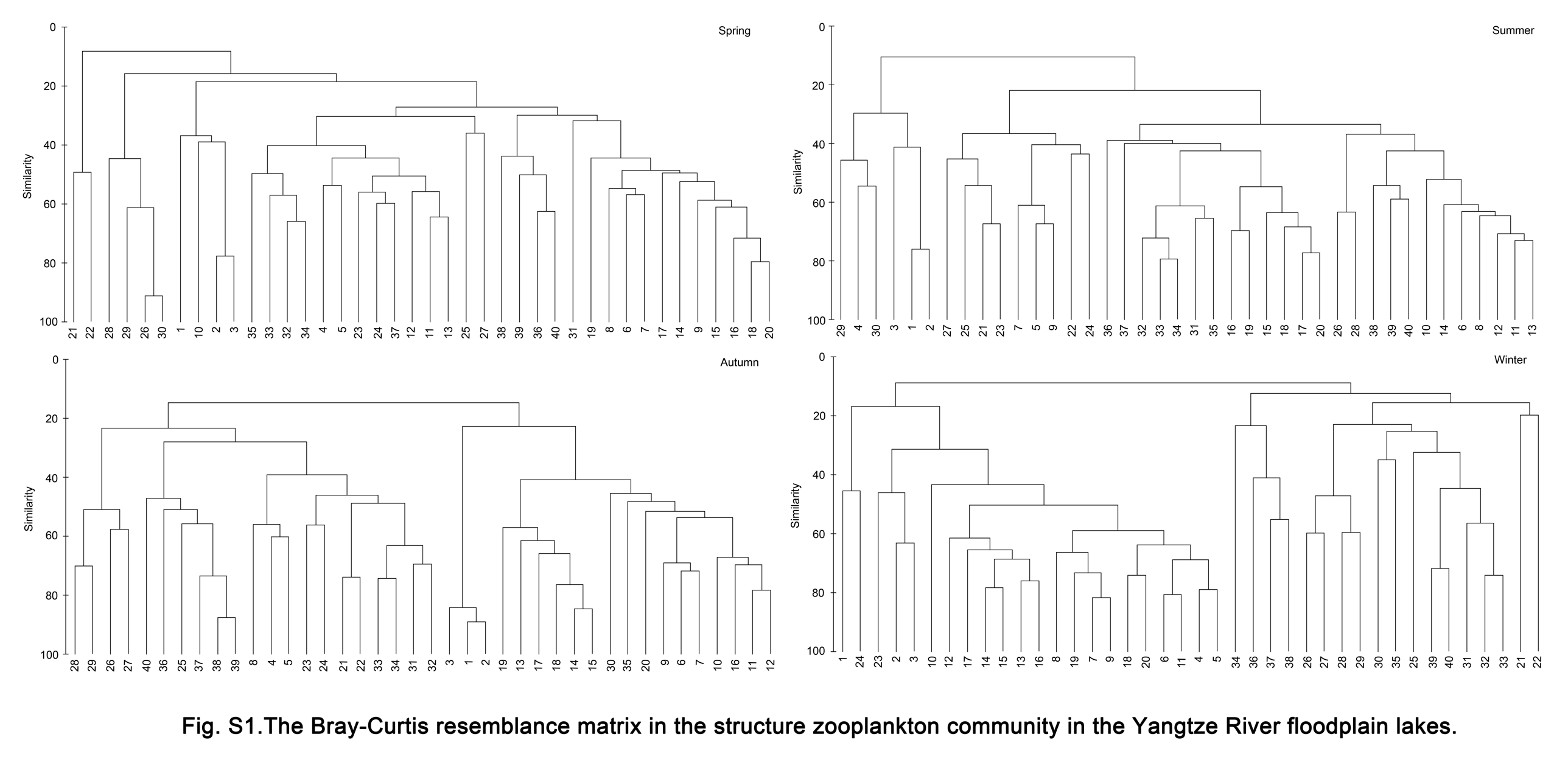

Supplement: Supplementary file 1 — Fig S1 [file ECE3-11-17885-s001.jpg]
