# Supplementary material for: Changes and drivers of zooplankton diversity patterns in the middle reach of Yangtze River floodplain lakes, China
Source: Ecol Evol. 2021 Dec 15;11(24):17885–900. doi: 10.1002/ece3.8353 (PMC8717274; doi:10.1002/ece3.8353)
Supplement: Supplementary file 2 — Table S1 [file ECE3-11-17885-s003.docx]

**TABLE S1** Environmental factors and habitat characteristics in the Yangtze River floodplain lakes (mean value ± SD). YR: the middle reach of the Yangtze River; TJ: the connected river channel of Poyang Lake; ML: the main lake area of Poyang Lake; NJ: Nanjishan Lake; JS: Junshan Lake; QL: Qinglan Lake; SH: Shahu Lake; DO: dissolved oxygen (mg/L); pH: hydrogen ions; Sal: salinity (mg/L); TURB: turbidity (NTU); T: water temperature (°C); Chl-a: chlorophyll-a (mg/L); V: water velocity (m/s); WD: water depth (m); TN: Total nitrogen (mg/L); TP: total phosphorus (mg/L).

|  | YR | TJ | ML | NJ | JS | QL | SH |
| --- | --- | --- | --- | --- | --- | --- | --- |
| WD (m) | 13.83 ± 4.06 | 8.96 ± 1.31 | 5.9 ± 1.01 | 1.67 ± 0.45 | 5.03 ± 0.47 | 3.45 ± 1.22 | 1.75 ± 0.43 |
| V (m/s) | 0.38 ± 0.09 | 0.31 ± 0.04 | 0.21 ± 0.04 | 0.10 ± 0.02 | 0.15 ± 0.03 | 0.17 ± 0.04 | 0.11 ± 0.22 |
| Turb (NTU^+^) | 13.5 ± 4.39 | 26.5 ± 6.15 | 13.16 ± 0.75 | 73.72 ± 21.83 | 6.55 ± 3.68 | 30.67 ± 18.16 | 50.95 ± 26.64 |
| T(℃) | 19.85 ± 2.96 | 19.72 ± 3.63 | 19.63 ± 4.08 | 19.31 ± 3.95 | 20.29 ± 4.33 | 21.43 ± 3.94 | 19.46 ± 4.46 |
| Sal (mg/L) | 0.13 ± 0.03 | 0.05 ± 0.01 | 0.04 ± 0.01 | 0.05 ± 0.01 | 0.08 ± 0.03 | 0.06 ± 0.01 | 0.05 ± 0.02 |
| DO (mg/L) | 8.75 ± 0.11 | 8.71 ± 0.05 | 8.16 ± 0.25 | 7.56 ± 0.23 | 8.46 ± 0.31 | 7.66 ± 0.38 | 8.72 ± 0.74 |
| Chl-*a* (μg/L) | 5.11 ± 1.56 | 17.08 ± 1.99 | 16.69 ± 4.08 | 25.28 ± 4.97 | 10.28 ± 3.72 | 37.98 ± 11.88 | 17.61 ± 1.29 |
| pH | 6.8 ± 0.31 | 6.67 ± 0.11 | 6.83 ± 0.12 | 7.21 ± 0.25 | 7.32 ± 0.41 | 7.09 ± 0.27 | 6.99 ± 0.23 |
| TN (mg/L) | 1.92 ± 0.03 | 1.75 ± 0.12 | 1.65 ± 0.17 | 1.74 ± 0.2 | 1.98 ± 0.48 | 0.92 ± 0.12 | 1.88 ± 0.59 |
| TP (mg/L) | 0.15 ± 0.03 | 0.16 ± 0.02 | 0.16 ± 0.01 | 0.18 ± 0.01 | 0.22 ± 0.05 | 0.11 ± 0.01 | 0.22 ± 0.04 |
| Area (km^2^) | 25 | 270 | 3210 | 3.52 | 213 | 112 | 14 |
| River/Lake | River | Lake | Lake | Lake | Lake | Lake | Lake |
| Number of macrophytes | Low | Low | High | High | Low | Middle | Middle |
| Substrate types | Sand | Hard mud, sand, silt | Hard mud, sand, silt | Silt | Silt | Silt, sand | Silt |
| Anthropogenic disturbances | Sand mining; industrial pollution; urban development | Sand mining; drought; urban development | Sand mining;  eutrophication;  overfishing;  drought | Eutrophication; aquaculture | Aquaculture | Aquaculture; eutrophication; overfishing | Drought |
