# Supplementary material for: Changes and drivers of zooplankton diversity patterns in the middle reach of Yangtze River floodplain lakes, China
Source: Ecol Evol. 2021 Dec 15;11(24):17885–900. doi: 10.1002/ece3.8353 (PMC8717274; doi:10.1002/ece3.8353)
Supplement: Supplementary file 3 — Table S2 [file ECE3-11-17885-s002.docx]

**TABLE S2** Distribution and composition of zooplankton in the Yangtze River floodplain lakes. 1 account for the presence of the species as native in the region, 0 means the species is absent from the region. YR: the middle reach of the Yangtze River; TJ: the connected river channel of Poyang Lake; ML: the main lake area of Poyang Lake; NJ: Nanjishan Lake; JS: Junshan Lake; QL: Qinglan Lake; SH: Shahu Lake.

|  | YR | TJ | ML | NJ | JS | QL | SH |
| --- | --- | --- | --- | --- | --- | --- | --- |
| *Rolaria neplunia* | 1 | 1 | 1 | 1 | 1 | 1 | 1 |
| *Rotaria rotatoria* | 1 | 1 | 1 | 1 | 0 | 1 | 1 |
| *Dicranophorus forcipatus* | 0 | 0 | 1 | 1 | 0 | 0 | 1 |
| *Dicranophorus lvtkeni* | 0 | 0 | 1 | 1 | 0 | 0 | 1 |
| *Lepadella apsida* | 0 | 0 | 1 | 1 | 0 | 1 | 0 |
| *Lepadella patella* | 1 | 1 | 1 | 1 | 1 | 1 | 1 |
| *Trichotria pocillum* | 1 | 1 | 1 | 1 | 1 | 1 | 1 |
| *Trichotria truncata* | 0 | 0 | 0 | 1 | 0 | 1 | 0 |
| *Trichotria tetractis* | 1 | 1 | 1 | 1 | 0 | 1 | 1 |
| *Brachiomus caudatu* | 0 | 0 | 1 | 1 | 0 | 0 | 0 |
| *Brachionus urceus* | 0 | 1 | 1 | 1 | 0 | 1 | 1 |
| *Brachionus angularis* | 1 | 1 | 1 | 1 | 1 | 1 | 1 |
| *Brachionus calyciflorus* | 1 | 1 | 1 | 1 | 1 | 1 | 1 |
| *Brachionus forficula* | 1 | 1 | 1 | 1 | 1 | 1 | 1 |
| *Brachionus budapestiensis* | 1 | 1 | 1 | 1 | 1 | 1 | 1 |
| *Brachionus leydigi* | 0 | 1 | 1 | 1 | 0 | 0 | 1 |
| *Brachionus quadridentatus* | 1 | 1 | 1 | 1 | 1 | 1 | 1 |
| *Brachionus forcatu* | 1 | 1 | 1 | 1 | 1 | 1 | 1 |
| *Brachionus capsuliforus* | 0 | 0 | 0 | 1 | 0 | 0 | 0 |
| *Brachionus diversicornis* | 1 | 1 | 1 | 1 | 1 | 1 | 1 |
| *Mytilina ventralis* | 0 | 0 | 0 | 1 | 0 | 1 | 0 |
| *Euchlanis dilatata* | 1 | 1 | 1 | 1 | 1 | 1 | 1 |
| *Anuraeopsis fissa* | 1 | 1 | 1 | 1 | 1 | 1 | 1 |
| *Keratella valga* | 1 | 1 | 1 | 1 | 1 | 1 | 1 |
| *Keratella quadrata* | 0 | 1 | 0 | 1 | 0 | 1 | 1 |
| *Keratella cochlearis* | 1 | 1 | 1 | 1 | 1 | 1 | 1 |
| *Keratella ticinensis* | 0 | 0 | 0 | 1 | 0 | 0 | 0 |
| *Plalyias qualricon*is | 0 | 1 | 1 | 1 | 1 | 0 | 0 |
| *Playias militaris* | 1 | 1 | 1 | 1 | 1 | 1 | 1 |
| *Notholca squamula* | 0 | 0 | 0 | 1 | 0 | 0 | 0 |
| *Notholca labis* | 1 | 1 | 0 | 1 | 0 | 0 | 1 |
| *Rhinoglena frontalis* | 0 | 0 | 0 | 0 | 0 | 0 | 1 |
| *Epiphanes senta* | 0 | 0 | 0 | 1 | 0 | 1 | 1 |
| *Lecane niothis* | 0 | 1 | 1 | 1 | 0 | 0 | 1 |
| *Lecane ungulata* | 1 | 1 | 1 | 1 | 1 | 1 | 1 |
| *Lecane flealls* | 0 | 0 | 0 | 1 | 0 | 0 | 0 |
| *Lecane luna* | 1 | 1 | 1 | 1 | 1 | 1 | 1 |
| *Lecane inermis* | 0 | 0 | 0 | 1 | 0 | 0 | 0 |
| *Moonostyla bulla* | 1 | 1 | 1 | 1 | 1 | 1 | 1 |
| *Monostyla elachis* | 0 | 0 | 0 | 1 | 0 | 0 | 0 |
| *Lecane luna* | 0 | 1 | 1 | 1 | 0 | 0 | 1 |
| *Monosty cornuta* | 1 | 0 | 1 | 1 | 0 | 0 | 1 |
| *Monostyla quadridentata* | 0 | 0 | 1 | 1 | 0 | 0 | 0 |
| *Monostyla* sp. | 1 | 1 | 1 | 1 | 0 | 1 | 1 |
| *Asplanchna brightwel* | 0 | 1 | 1 | 1 | 1 | 1 | 1 |
| *Asplanchna priodonta* | 1 | 1 | 1 | 1 | 1 | 1 | 1 |
| *Asplanchna girodi* | 1 | 1 | 1 | 1 | 1 | 1 | 1 |
| *Asplanchnopus multiceps* | 0 | 1 | 1 | 1 | 1 | 1 | 1 |
| *Notommata tripus* | 0 | 0 | 0 | 1 | 0 | 0 | 1 |
| *Notommata copeus* | 0 | 0 | 1 | 1 | 0 | 0 | 0 |
| *Eothinia elongata* | 1 | 0 | 1 | 1 | 1 | 1 | 1 |
| *Cephalodella exigna* | 1 | 1 | 1 | 1 | 1 | 1 | 1 |
| *Cephalodella gibba* | 1 | 1 | 1 | 1 | 1 | 1 | 1 |
| *Scaridum longicaudu*m | 1 | 1 | 1 | 1 | 1 | 1 | 1 |
| *Gastropus hyptopus* | 1 | 1 | 1 | 1 | 0 | 1 | 1 |
| *Ascomorpha ecaudis* | 0 | 1 | 0 | 1 | 1 | 0 | 1 |
| *Ascomorpha saltans* | 1 | 1 | 1 | 1 | 1 | 1 | 1 |
| *Ascomorpha ovalis* | 0 | 1 | 1 | 1 | 1 | 1 | 1 |
| *Trichocerca weberi* | 1 | 1 | 1 | 1 | 1 | 1 | 1 |
| *Trichocerca bicristata* | 0 | 1 | 1 | 1 | 0 | 1 | 0 |
| *Trichocerca rattus* | 0 | 1 | 1 | 1 | 0 | 1 | 1 |
| *Trichocerca cylindrical* | 1 | 1 | 1 | 1 | 1 | 1 | 1 |
| *Trichocerca capucina* | 1 | 1 | 1 | 1 | 1 | 1 | 1 |
| *Trichocerca longisrta* | 1 | 1 | 1 | 1 | 1 | 1 | 1 |
| *Trichocerca longiseta* | 1 | 1 | 1 | 1 | 1 | 1 | 1 |
| *Trichocerca lophoessa* | 1 | 1 | 1 | 1 | 1 | 1 | 1 |
| *Polyarthra trigla* | 0 | 1 | 1 | 1 | 1 | 1 | 1 |
| *Polyarthra dolichoptera* | 1 | 1 | 1 | 1 | 1 | 1 | 1 |
| *Polyarthra unlgaris* | 1 | 1 | 1 | 1 | 1 | 1 | 1 |
| *Polyarthra eurypetera* | 1 | 1 | 0 | 1 | 0 | 0 | 1 |
| *Macrochaetus* | 0 | 0 | 0 | 1 | 0 | 0 | 0 |
| *Synchacta atylata* | 0 | 1 | 1 | 1 | 0 | 0 | 0 |
| *Synchacta grandis* | 1 | 1 | 1 | 1 | 1 | 1 | 1 |
| *Synchacta tremula* | 1 | 1 | 1 | 1 | 1 | 1 | 1 |
| *Synchacta pectinata* | 1 | 1 | 1 | 1 | 1 | 1 | 1 |
| *Synchaeta oblonga* | 1 | 1 | 1 | 1 | 1 | 1 | 1 |
| *Synchacta cylonga* | 0 | 1 | 1 | 1 | 0 | 1 | 1 |
| *Ploesoma hudsoni* | 1 | 1 | 1 | 1 | 1 | 1 | 1 |
| *Ploesoma truncatum* | 0 | 1 | 0 | 1 | 0 | 0 | 1 |
| *Testudinella patina* | 1 | 1 | 1 | 1 | 1 | 1 | 0 |
| *Hexarthra mira* | 1 | 1 | 1 | 1 | 1 | 1 | 1 |
| *Filinia longiseta* | 1 | 1 | 1 | 1 | 1 | 1 | 1 |
| *Filinia passa* | 0 | 1 | 0 | 1 | 0 | 1 | 1 |
| *Tetramastix opoliensis* | 0 | 1 | 1 | 1 | 0 | 1 | 0 |
| *Conochiloides dossuarius* | 0 | 0 | 0 | 1 | 0 | 0 | 0 |
| *Conochilus unicornis* | 1 | 1 | 1 | 1 | 0 | 0 | 1 |
| *Collotheea pelagica* | 1 | 1 | 1 | 1 | 1 | 0 | 0 |
| *Collotheea mutabilis* | 0 | 1 | 1 | 1 | 1 | 0 | 0 |
| *Leptodora kindtii* | 0 | 0 | 0 | 1 | 1 | 0 | 1 |
| *Sida erystallina* | 1 | 1 | 1 | 1 | 1 | 1 | 0 |
| *Diaphanosoma leuchtenbergianum* | 1 | 1 | 1 | 1 | 1 | 1 | 1 |
| *Diaphanosoma brachyurum* | 1 | 1 | 1 | 1 | 1 | 1 | 1 |
| *Diaphnia cucullata* | 1 | 1 | 1 | 1 | 1 | 1 | 1 |
| *Daphnia* | 1 | 0 | 0 | 1 | 1 | 1 | 0 |
| *Ceriodaphnia quadrangula* | 1 | 1 | 1 | 1 | 1 | 1 | 1 |
| *Ceriodaphnia cornigera* | 1 | 0 | 0 | 0 | 0 | 0 | 0 |
| *Scapholeberis kingi* | 0 | 0 | 0 | 0 | 0 | 1 | 0 |
| *Moina micrura* | 1 | 1 | 1 | 1 | 1 | 1 | 1 |
| *Bosmina longirostris* | 1 | 1 | 1 | 1 | 1 | 1 | 1 |
| *Bosmina fatalis* | 0 | 1 | 0 | 1 | 0 | 0 | 0 |
| *Bosnina coregoni* | 1 | 1 | 1 | 1 | 1 | 1 | 1 |
| *Bosminopsis deitersi* | 1 | 1 | 1 | 1 | 1 | 1 | 1 |
| *Ilyocryptus sordidus* | 0 | 1 | 0 | 1 | 0 | 0 | 1 |
| *Alona rectangula* | 1 | 1 | 1 | 1 | 1 | 1 | 1 |
| *Alona guttata* | 0 | 1 | 1 | 1 | 0 | 1 | 1 |
| *Pleuroxus hamulatus* | 0 | 1 | 1 | 1 | 0 | 1 | 0 |
| *Pleuroxus laevis* | 0 | 0 | 0 | 1 | 0 | 0 | 0 |
| *Alonella excisa* | 0 | 1 | 0 | 1 | 0 | 0 | 0 |
| *Alonella rostrata* | 0 | 0 | 0 | 1 | 0 | 0 | 0 |
| *Chydorus sphaericus* | 1 | 0 | 1 | 1 | 1 | 1 | 1 |
| *Chydorus gibbus* | 0 | 1 | 1 | 1 | 0 | 0 | 0 |
| *Camptocercus rectirostris* | 0 | 0 | 0 | 1 | 0 | 0 | 0 |
| Copepods nauplii | 1 | 1 | 1 | 1 | 1 | 1 | 1 |
| *Sinocalanus dorrii* | 1 | 1 | 1 | 1 | 1 | 1 | 1 |
| *Schmackeria forbesi* | 1 | 1 | 1 | 1 | 1 | 0 | 1 |
| *Neodiaptomus schmackeri* | 1 | 1 | 1 | 0 | 1 | 1 | 1 |
| *Eodiaptomus sinensi*s | 0 | 0 | 0 | 0 | 0 | 1 | 0 |
| *Diaptomidae incongruens* | 1 | 1 | 1 | 1 | 1 | 1 | 1 |
| *Limnocletodes behningi* | 0 | 1 | 1 | 1 | 1 | 0 | 1 |
| *Nitorca lacustris* | 1 | 1 | 1 | 1 | 0 | 1 | 0 |
| *Limnoithona sinensis* | 1 | 1 | 1 | 1 | 0 | 1 | 0 |
| *Macrocyclops albidus* | 1 | 1 | 1 | 0 | 1 | 1 | 0 |
| *Cyclopidae vicinus* | 1 | 1 | 1 | 1 | 1 | 1 | 1 |
| *Mesocyclops leuckarti* | 1 | 1 | 1 | 1 | 1 | 1 | 1 |
| *Microcyclops varicans* | 1 | 1 | 1 | 1 | 1 | 1 | 1 |
| *Thermocyclops kawamurai* | 1 | 1 | 1 | 0 | 0 | 1 | 0 |
| *Thermocyclops taihokuensis* | 1 | 1 | 1 | 1 | 1 | 1 | 1 |
| *Eucyclops speratus* | 1 | 1 | 1 | 1 | 0 | 1 | 1 |
